# The link between adjacent codon pairs and mRNA stability

**DOI:** 10.1186/s12864-017-3749-8

**Published:** 2017-05-10

**Authors:** Yuriko Harigaya, Roy Parker

**Affiliations:** Department of Chemistry and Biochemistry, Howard Hughes Medical Institute, University of Colorado Boulder, Boulder, CO 80303 USA

**Keywords:** Codon pair, mRNA translation, mRNA stability

## Abstract

**Background:**

Evidence in diverse organisms suggests that codon optimality is a major determinant of mRNA translation and degradation. Codon optimality is thought to act by modulating the efficiency of ribosome elongation. In *Saccharomyces cerevisiae*, a recent study has identified 17 adjacent codon pairs that mediate strong inhibition of translation elongation. However, relationships between the inhibitory codon pairs and other aspects of gene expression are unknown.

**Results:**

To gain insights into how the inhibitory codon pairs may affect aspects of gene expression, we utilized existing datasets to conduct genome-scale analyses in *S. cerevisiae*. Our analysis revealed the following points. First, the inhibitory codon pairs are significantly associated with faster mRNA decay. The association is not solely due to the content of nucleotides, individual codons, or dipeptides encoded by the inhibitory codon pairs. Second, the inhibitory codon pairs cannot fully explain the previously known relationship of codon optimality with mRNA stability, suggesting that optimality of individual codons and properties of adjacent codon pairs both contribute to gene regulation. Finally, although the inhibitory codon pairs are associated with slower mRNA synthesis and protein instability, the associations can be attributed to usage bias in individual codons.

**Conclusions:**

This study suggests an association of inhibitory codon pairs with mRNA stability and thus another layer of complexity in the codon-mediated gene regulation.

**Electronic supplementary material:**

The online version of this article (doi:10.1186/s12864-017-3749-8) contains supplementary material, which is available to authorized users.

## Background

mRNA degradation is a critical step in gene expression, and the decay rates of individual mRNAs can vary over two orders of magnitude. Differences in the decay rates of individual mRNAs can be specified by several features of the mRNAs. They include sequence motifs that are recognized by *trans*-acting factors, such as microRNAs and RNA-binding proteins. Strikingly, in many of these cases, the *trans*-acting factors can also decrease translation initiation, which suggests a tight coupling of translation initiation and mRNA degradation [1].

Perturbations of translation elongation can also affect mRNA degradation. For example, strong blocks to translation elongation trigger endonucleolytic cleavage of the mRNA in a process called no-go decay [2, 3]. However, until a recent study by Coller and colleagues [4], it was not appreciated that subtle differences in the rates of translation elongation due to specific codons, which can be indicated by “codon optimality,” would contribute in a general manner to defining mRNA decay rates. The general model is that “optimal” codons, which are decoded efficiently, are associated with mRNA stability, whereas “nonoptimal” codons, which are decoded slowly, are associated with mRNA instability. The study in *Saccharomyces cerevisiae* by Coller and colleagues was followed by multiple studies in diverse organisms arguing that codon-mediated mRNA decay is a broadly conserved phenomenon [5–8]. Moreover, data suggest that the codon-mediated mRNA decay is accompanied by a reduction in translation efficiency, defined as protein synthesis rates per mRNA [4, 7, 9], which may result from a decrease in translation initiation rate [10].

A more recent study in *S. cerevisiae* by Grayhack, Fields, and colleagues has demonstrated that adjacent codon pairs also influence translation in a manner distinct from their individual constituent codons [11]. This suggests that optimality of individual codons does not solely define the relationship between codon composition and translation efficiency. Specifically, via a large-scale flow cytometry analysis using GFP reporter variants, the authors have identified 17 adjacent codon pairs that act inhibitory on protein expression. A series of subsequent analyses using the reporter system suggest several important aspects of the inhibition of protein expression mediated by the codon pairs [11]. First, the inhibition is mediated by the codon pairs themselves rather than by the corresponding hexanucleotide sequences, individual constituent codons, or encoded dipeptides. Second, the inhibition occurs during translation elongation largely depending on wobble decoding of either the 5’ and/or 3’ codon. Third, in some instances, the inhibitory codon pairs are associated with a reduction in mRNA abundance consistent with the codon pairs eliciting mRNA instability.

The findings of additional analyses of genomic data imply that the inhibitory codon pairs may be relevant to the regulation of natural endogenous genes [11]. First, ribosome occupancy is substantially elevated at most of the inhibitory codon pairs, which suggests that the codon pairs slow translation elongation. Second, the inhibitory codon pairs are enriched in genes whose mRNA abundance is low. Third, translation efficiency, as assessed by protein abundance per mRNA, of genes containing at least one of the 17 inhibitory codon pairs are significantly lower than that of genes lacking them. This tendency persists even when the analysis is controlled for usage bias in individual codons.

The observation that inhibitory codon pairs can reduce translation elongation rates and affect mRNA levels raises the possibility that the effects of codon optimality on gene expression parameters could be explained by the presence of inhibitory codon pairs [11]. Alternatively, both overall “codon optimality” and inhibitory codon pairs could act in a similar manner to slow elongation and thereby both contribute to changes in gene expression as a downstream read-out of translation elongation rates.

In this work, we examined relationships between the inhibitory codon pairs and additional aspects of gene expression on a genomic scale in *S. cerevisiae*. Our analysis revealed consistent associations of the inhibitory codon pairs with fast mRNA decay in multiple RNA kinetic datasets. The association persisted after controlling for the content of nucleotides, optimality of codons contained by the mRNA, and the content of dipeptides encoded by the inhibitory pairs, suggesting that the link between the inhibitory codon pairs and mRNA decay rates is not solely due to effects of the covariates. Additional analyses suggest that the presence of the inhibitory codon pairs cannot fully explain the relationship of codon optimality with mRNA stability or translation efficiency. Overall, our study has revealed novel aspects concerning the relationship between adjacent codon pairs and the regulation of gene expression.

## Results

### The inhibitory codon pairs are associated with inefficient synthesis and instability of mRNA

It has been shown that genes with the inhibitory codon pairs are enriched in genes whose mRNA abundance is low [11]. Since mRNA abundance is determined by rates of mRNA synthesis and decay, we examined whether the previously identified 17 inhibitory codon pairs (1) are associated with mRNA synthesis and/or decay. For this purpose, we computed Spearman’s and Kendall’s correlation coefficients between the outcome (i.e., mRNA synthesis/decay rates) and predictor (i.e., the inhibitory codon pairs) variables (Methods). As an outcome, we used RNA kinetic values from two previous studies by Cramer and colleagues and one by Gresham and colleagues (the “Cramer 1”, “Cramer 2”, and “Gresham” datasets) [12–14]. We selected these datasets because the metabolic labeling method, which was used in the studies, has been suggested to be less intrusive than others [12, 14, 15]. To examine whether conclusions from the analyses are independent from the methods of RNA kinetic measurements, we also analyzed a dataset from a previous study by Coller and colleagues (the “Coller” dataset), which was generated via transcription inhibition [4]. As a predictor, we used two types of variables, the fraction of inhibitory codon pairs contained in mRNA and a binary variable to indicate the presence or absence of at least one of the 17 inhibitory codon pairs. In this analysis, we aggregated the inhibitory codon pairs rather than analyzing them individually since some of them occur very infrequently in the coding sequences (Table 1). For each dataset, the number of genes containing at least one of the inhibitory codon pairs is shown in Additional file 1: Table S1. Although we computed *P* values by methods described by Kim [16] as well as by permutation tests (Methods), we primarily used permutation *P* values with a significant threshold of *P* < 0.05 for hypothesis testing.

**Table 1:** The 17 inhibitory codon pairs

	Number of occurrences	Number of genes
AGGCGA	113	113
AGGCGG	89	87
ATACGA	248	236
ATACGG	140	134
CGAATA	214	203
CGACCG	16	16
CGACGA	14	13
CGACGG	18	17
CGACTG	76	75
CGAGCG	31	30
CTCCCG	53	53
CTGATA	532	475
CTGCCG	179	169
CTGCGA	141	137
GTACCG	263	247
GTACGA	188	181
GTGCGA	71	70

Shown are the nucleotide sequences of the inhibitory codon pairs, the number of occurrences of each inhibitory codon pair, and the number of genes that contain at least one of the inhibitory codon pairs

**Fig. 1:**
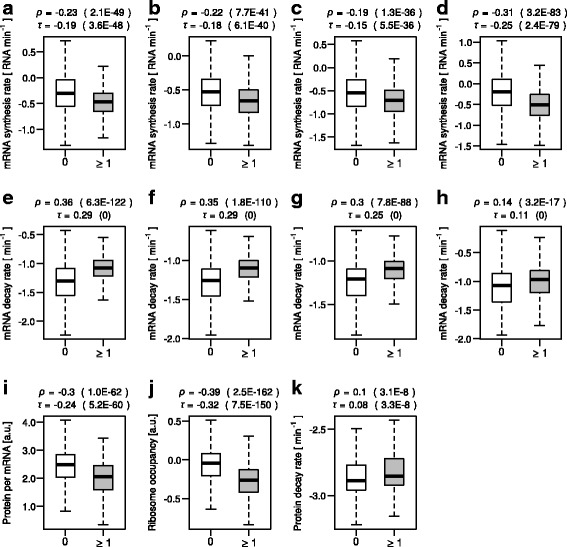
Associations of the inhibitory codon pairs with synthesis and decay of mRNA and protein. **a** Boxplot comparing mRNA synthesis rates in the “Cramer 1” data (in log10 scale) between genes containing at least one of the 17 inhibitory codon pairs (≥1) and those without them (0). Shown on the top are the Spearman’s and Kendall’s correlation coefficients and *P* values (parenthesis) to assess an association of the presence (1) and absence (0) of the inhibitory codon pairs with mRNA synthesis rate. **b** Same as (**a**) but for the “Cramer 2” data. **c** Same as (**a**) but for the “Gresham” data. **d** Same as (**a**) but for the “Coller” data. **e** Same as (**a**) but for mRNA decay rate. **f** Same as (**e**) but for the “Cramer 2” data. **g** Same as (**e**) but for the “Gresham” data. **h** Samea s (**e**) but for the “Coller” data. **i** Same as (**a**) but for protein abundance per mRNA. **j** Same as (**a**) but for ribosome occupancy. **k** Same as (**a**) but for protein decay rates

The analysis of the four datasets consistently led to two associations. First, the fraction of the inhibitory codon pairs as well as the binary indicator of the presence thereof was associated with slow mRNA synthesis rates, which was suggested by negative correlations (Fig. 1a-d, Table 2A, and B). Second, both variables representing the content of the inhibitory codon pairs were also associated with fast mRNA decay rates, which was suggested by positive correlations (Fig. 1e-h, Table 2A, and B).

**Table 2:** Test for associations between the inhibitory codon pairs and various gene expression variables

(A) Correlation based on the fraction of the inhibitory codon pairs						
	Spearman			Kendall		
	*ρ*	*P* value	Permutation *P* value	*τ*	*P* value	Permutation *P* value
mRNA synthesis rate (Cramer 1)	−0.23	5.1E-51	1.0E-04	−0.18	9.7E-51	1.0E-04
mRNA synthesis rate (Cramer 2)	−0.20	1.6E-36	1.0E-04	−0.15	3.6E-36	1.0E-04
mRNA synthesis rate (Gresham)	−0.22	1.6E-50	1.0E-04	−0.17	5.6E-52	1.0E-04
mRNA synthesis rate (Coller)	−0.28	5.5E-68	1.0E-04	−0.21	2.1E-63	1.0E-04
mRNA decay rate (Cramer 1)	0.37	1.3E-133	1.0E-04	0.29	0.0E + 00	1.0E-04
mRNA decay rate (Cramer 2)	0.35	1.2E-109	1.0E-04	0.26	0.0E + 00	1.0E-04
mRNA decay rate (Gresham)	0.25	2.9E-59	1.0E-04	0.19	0.0E + 00	1.0E-04
mRNA decay rate (Coller)	0.17	5.1E-25	1.0E-04	0.13	0.0E + 00	1.0E-04
Protein per mRNA	−0.31	2.9E-70	1.0E-04	−0.24	1.4E-67	1.0E-04
Ribosome occupancy	−0.34	1.2E-120	1.0E-04	−0.25	8.3E-112	1.0E-04
Protein decay rate	0.10	1.7E-08	1.0E-04	0.08	1.4E-08	1.0E-04
(B) Correlation based on the presence/absence of the inhibitory codon pairs						
	Spearman			Kendall		
	*ρ*	*P* value	Permutation *P* value	*τ*	*P* value	Permutation *P* value
mRNA synthesis rate (Cramer 1)	−0.23	2.1E-49	1.0E-04	−0.19	3.6E-48	1.0E-04
mRNA synthesis rate (Cramer 2)	−0.22	7.7E-41	1.0E-04	−0.18	6.1E-40	1.0E-04
mRNA synthesis rate (Gresham)	−0.19	1.3E-36	1.0E-04	−0.15	5.5E-36	1.0E-04
mRNA synthesis rate (Coller)	−0.31	3.2E-83	1.0E-04	−0.25	2.4E-79	1.0E-04
mRNA decay rate (Cramer 1)	0.36	6.3E-122	1.0E-04	0.29	0.0E + 00	1.0E-04
mRNA decay rate (Cramer 2)	0.35	1.8E-110	1.0E-04	0.29	0.0E + 00	1.0E-04
mRNA decay rate (Gresham)	0.30	7.8E-88	1.0E-04	0.25	0.0E + 00	1.0E-04
mRNA decay rate (Coller)	0.14	3.2E-17	1.0E-04	0.11	0.0E + 00	1.0E-04
Protein per mRNA	−0.30	1.0E-62	1.0E-04	−0.24	5.2E-60	1.0E-04
Ribosome occupancy	−0.39	2.5E-162	1.0E-04	−0.32	7.5E-150	1.0E-04
Protein decay rate	0.10	3.1E-08	1.0E-04	0.08	3.3E-08	1.0E-04
(C) Partial correlation based on the fraction of the inhibitory codon pairs						
	Spearman			Kendall		
	*ρ*	*P* value	Permutation *P* value	*τ*	*P* value	Permutation *P* value
mRNA synthesis rate (Cramer 1)	−0.01	5.1E-01	2.6E-01	−0.06	8.4E-09	1.0E-04
mRNA synthesis rate (Cramer 2)	−0.05	8.7E-04	3.0E-04	−0.08	1.7E-13	1.0E-04
mRNA synthesis rate (Gresham)	−0.04	1.2E-02	5.9E-03	−0.07	3.0E-11	1.0E-04
mRNA synthesis rate (Coller)	−0.02	2.3E-01	1.1E-01	−0.08	7.3E-13	1.0E-04
mRNA decay rate (Cramer 1)	0.14	2.1E-18	1.0E-04	0.15	2.4E-48	1.0E-04
mRNA decay rate (Cramer 2)	0.15	1.2E-20	1.0E-04	0.16	9.8E-49	1.0E-04
mRNA decay rate (Gresham)	0.07	9.0E-06	1.0E-04	0.10	7.9E-22	1.0E-04
mRNA decay rate (Coller)	0.06	2.0E-04	1.0E-04	0.07	6.2E-10	1.0E-04
Protein per mRNA	−0.07	4.9E-05	1.0E-04	−0.11	7.3E-20	1.0E-04
Ribosome occupancy	−0.06	8.8E-05	1.0E-04	−0.11	1.3E-30	1.0E-04
Protein decay rate	−0.01	6.4E-01	3.3E-01	0.02	7.7E-02	6.2E-02
(D) Partial correlation based on the presence/absence of the inhibitory codon pairs						
	Spearman			Kendall		
	*ρ*	*P* value	Permutation *P* value	*τ*	*P* value	Permutation *P* value
mRNA synthesis rate (Cramer 1)	0.00	8.0E-01	4.1E-01	−0.06	5.7E-09	1.0E-04
mRNA synthesis rate (Cramer 2)	−0.05	3.3E-03	1.3E-03	−0.09	1.2E-15	1.0E-04
mRNA synthesis rate (Gresham)	−0.03	5.0E-02	2.7E-02	−0.06	1.3E-09	1.0E-04
mRNA synthesis rate (Coller)	−0.02	1.9E-01	9.6E-02	−0.10	2.6E-18	1.0E-04
mRNA decay rate (Cramer 1)	0.13	2.8E-16	1.0E-04	0.16	1.1E-50	1.0E-04
mRNA decay rate (Cramer 2)	0.14	8.6E-19	1.0E-04	0.17	2.7E-57	1.0E-04
mRNA decay rate (Gresham)	0.07	2.2E-05	1.0E-04	0.12	8.3E-29	1.0E-04
mRNA decay rate (Coller)	0.04	7.5E-03	3.8E-03	0.06	5.9E-08	1.0E-04
Protein per mRNA	−0.07	6.3E-05	1.0E-04	−0.12	3.2E-23	1.0E-04
Ribosome occupancy	−0.06	1.6E-05	2.0E-04	−0.14	3.7E-44	1.0E-04
Protein decay rate	0.00	7.8E-01	3.9E-01	0.02	3.6E-02	5.0E-02

(A) Spearman’s and Kendall’s correlation coefficients to assess an association between the fraction of the inhibitory codon pairs and various gene expression variables. *P* values obtained according to Kim [16] and those based on permutation tests are shown. (B) Same as (A) but for the presence/absence of the inhibitory codon pairs. (C) Spearman’s and Kendall’s partial correlation coefficients controlled for GC content, tAI, dipeptide content, coding length to assess an association between the fraction of the inhibitory codon pairs and various gene expression variables. (D) Same as (C) but for the presence/absence of the inhibitory codon pairs

In principle, the observed associations could be due to the inhibitory codon pairs or to other transcript features since several other transcript features were correlated with the content of the inhibitory codon pairs as well as with mRNA synthesis/decay rates in some of the datasets (Additional file 2: Table S2). These include guanine-cytosine (GC) content, tRNA adaptation index (tAI), which is a metric of codon optimality (Methods), the fraction of dinucleotides that are encoded by the inhibitory pairs, and the lengths of coding sequences. To evaluate contributions of these transcript features to the observed associations, we computed Spearman’s and Kendall’s partial correlation coefficients between the content of the inhibitory codon pairs and mRNA decay/synthesis rates controlling for these confounding factors. The analysis led to the following two points. First, for mRNA decay rates, the association remained significant when the analysis was individually controlled for GC content, codon optimality (tAI), dinucleotide content, and coding lengths with one exception where we analyzed a relationship between the presence/absence of the inhibitory codon pairs and mRNA decay rates in the “Coller” dataset controlling for codon optimality (tAI) using the Spearman’s method (Permutation *P* value = 0.13) (Additional file 3: Table S3). The association was significant across the analysis methods and datasets when the analysis was controlled for all the covariates (Table 2C and D). Second, for mRNA synthesis rates, the association was no longer consistent across the analysis methods/datasets when the analysis was controlled for codon optimality (tAI) or for all covariates (Table 2C, D, and Additional file 3: Table S3).

Overall, the results suggest an association of the inhibitory codon pairs with mRNA instability on a genomic scale, which appears to be, at least in part, independent of the content of nucleotides, individual constituent codons, and encoded dipeptides as well as of coding lengths. Although we also observed an association between the inhibitory codon pairs and mRNA synthesis rates, this could be attributed to usage bias in individual codons.

### The association between inhibitory codon pairs and mRNA instability is largely dependent on the correct reading frame

The simplest model is that inhibitory codon pairs correlate with mRNA decay rates due to their effects on translation elongation and would thus only correlate with mRNA decay rates when present in the proper reading frame. Alternatively, it remains possible that the hexanucleotides making up inhibitory codon pairs could affect mRNA decay rates directly. For example, one possibility is that a subset of the sequences might coincidentally match those recognized by *trans*-acting factors that promote mRNA decay. To address this issue, we examined an association of the occurrence of the hexanucleotide sequences in shifted reading frames as wells as in 3’ untranslated regions (3’ UTRs) with mRNA decay rates. In the former analysis, we computationally introduced frameshifts by one or two nucleotides to all ORFs and repeated otherwise the same correlation analyses as described above. In the latter analysis, to all ORFs, we assigned binary indicators to represent the presence/absence of at least one of the inhibitory codon pairs within 3’ UTR based on annotations from previous studies by Snyder and colleagues [17] and by Steinmetz and colleagues and [18].

The analyses led to the following points. First, the inhibitory codon pairs in the +1 frame were associated with fast mRNA decay rates across the analysis methods/datasets except for the “Coller” data (Additional file 4: Table S4). However, the association became inconsistent when GC content, codon optimality, the content of dipeptides encoded by the in-frame inhibitory codon pairs, and coding lengths were individually or simultaneously controlled for (Table 3 and Additional file 4: Table S4). Second, the hexanucleotide sequences in 3’ UTR were not consistently associated with mRNA decay rates (Table 4).

**Table 3:** Test for associations of the out-of-frame inhibitory codon pairs with mRNA decay rate, protein per mRNA, and ribosome occupancy

(A) Spearman’s partial correlation based on the fraction of the inhibitory codon pairs									
	Frame 0			Frame 1			Frame 2		
	*ρ*	*P* value	Perm. *P* value	*ρ*	*P* value	Perm. *P* value	*ρ*	*P* value	Perm. *P* value
mRNA decay rate (Cramer 1)	0.14	2.1E-18	1.0E-04	0.05	1.0E-03	7.0E-04	0.02	3.4E-01	1.7E-01
mRNA decay rate (Cramer 2)	0.15	1.2E-20	1.0E-04	0.02	2.4E-01	1.3E-01	0.03	3.8E-02	1.8E-02
mRNA decay rate (Gresham)	0.07	9.0E-06	1.0E-04	0.06	2.0E-04	2.0E-04	0.01	5.2E-01	2.6E-01
mRNA decay rate (Coller)	0.06	2.0E-04	1.0E-04	0.02	2.2E-01	1.1E-01	0.03	4.6E-02	2.4E-02
Protein per mRNA	−0.07	4.9E-05	1.0E-04	0.03	8.6E-02	4.3E-02	0.01	7.2E-01	3.6E-01
Ribosome occupancy	−0.06	8.8E-05	1.0E-04	0.02	2.0E-01	9.9E-02	0.01	5.2E-01	2.7E-01
(B) Kendall’s partial correlation based on the fraction of the inhibitory codon pairs									
	Frame 0			Frame 0			Frame 0		
	*τ*	*P* value	Perm. *P* value	*τ*	*P* value	Perm. *P* value	*τ*	*P* value	Perm. *P* value
mRNA decay rate (Cramer 1)	0.15	2.4E-48	1.0E-04	0.04	6.7E-04	1.0E-04	0.00	7.7E-01	3.8E-01
mRNA decay rate (Cramer 2)	0.16	9.8E-49	1.0E-04	0.02	2.5E-02	9.7E-03	0.02	7.1E-02	3.2E-02
mRNA decay rate (Gresham)	0.10	7.9E-22	1.0E-04	0.08	2.6E-13	1.0E-04	0.03	1.3E-02	4.0E-03
mRNA decay rate (Coller)	0.07	6.2E-10	1.0E-04	0.01	6.1E-01	3.1E-01	0.01	3.8E-01	2.0E-01
Protein per mRNA	−0.11	7.3E-20	1.0E-04	0.01	3.7E-01	1.7E-01	0.01	6.2E-01	3.1E-01
Ribosome occupancy	−0.11	1.3E-30	1.0E-04	−0.02	2.2E-02	3.9E-03	−0.01	4.6E-01	2.0E-01
(C) Spearman’s partial correlation based on the presence/absence of the inhibitory codon pairs									
	Frame 0			Frame 1			Frame 2		
	*ρ*	*P* value	Perm. *P* value	*ρ*	*P* value	Perm. *P* value	*ρ*	*P* value	Perm. *P* value
mRNA decay rate (Cramer 1)	0.13	2.8E-16	1.0E-04	0.04	1.1E-02	7.3E-03	0.00	8.7E-01	4.3E-01
mRNA decay rate (Cramer 2)	0.14	8.6E-19	1.0E-04	0.01	4.0E-01	2.0E-01	0.01	5.5E-01	2.7E-01
mRNA decay rate (Gresham)	0.07	2.2E-05	1.0E-04	0.06	2.9E-04	3.0E-04	0.01	6.0E-01	3.0E-01
mRNA decay rate (Coller)	0.04	7.5E-03	3.8E-03	0.02	1.7E-01	8.1E-02	0.02	2.7E-01	1.4E-01
Protein per mRNA	−0.07	6.3E-05	1.0E-04	0.03	1.6E-01	8.1E-02	0.00	9.9E-01	4.9E-01
Ribosome occupancy	−0.06	1.6E-05	2.0E-04	0.01	4.1E-01	2.0E-01	0.00	9.6E-01	4.8E-01
(D) Kendall’s partial correlation based on the presence/absence of the inhibitory codon pairs									
	Frame 0			Frame 0			Frame 0		
	*τ*	*P* value	Perm. *P* value	*τ*	*P* value	Perm. *P* value	*τ*	*P* value	Perm. *P* value
mRNA decay rate (Cramer 1)	0.16	1.1E-50	1.0E-04	0.03	1.2E-03	1.5E-03	0.00	1.0E + 00	4.9E-01
mRNA decay rate (Cramer 2)	0.17	2.7E-57	1.0E-04	0.04	8.8E-04	2.1E-03	0.02	6.1E-02	5.1E-02
mRNA decay rate (Gresham)	0.12	8.3E-29	1.0E-04	0.12	7.0E-31	1.0E-04	0.07	1.4E-10	1.0E-04
mRNA decay rate (Coller)	0.06	5.9E-08	1.0E-04	0.00	9.8E-01	4.9E-01	0.00	6.6E-01	3.5E-01
Protein per mRNA	−0.12	3.2E-23	1.0E-04	0.01	3.2E-01	2.0E-01	0.00	9.6E-01	4.8E-01
Ribosome occupancy	−0.14	3.7E-44	1.0E-04	−0.07	3.4E-11	1.0E-04	−0.05	2.6E-07	1.0E-04

(A) Spearman’s partial correlation coefficients controlled for GC content, tAI, dipeptide content, and coding length to assess an association between the fraction of hexanucleotide sequences corresponding to the inhibitory codon pairs in the 0, +1, and +2 frames and various gene expression variables. *P* values obtained according to Kim [16] and those based on permutation tests are shown. (B) Same as (A) but for Kendall’s partial correlation coefficients. (C) Same as (A) but for the presence/absence of the hexanucleotide sequences. (D) Same as (B) but for the presence/absence of the hexanucleotide sequences

**Table 4:** Test for associations of the inhibitory codon pairs in non-coding regions with mRNA decay rate, protein per mRNA, and ribosome occupancy

(A) Based on UTR annotations by Snyder and colleagues						
	Spearman			Kendall		
	*ρ*	*P* value	Permutation *P* value	*τ*	*P* value	Permutation *P* value
mRNA decay rate (Cramer 1)	0.03	6.3E-02	3.6E-02	0.02	6.3E-02	4.1E-02
mRNA decay rate (Cramer 2)	0.01	5.1E-01	2.6E-01	0.01	5.1E-01	2.7E-01
mRNA decay rate (Gresham)	0.00	9.6E-01	4.8E-01	0.00	9.6E-01	4.9E-01
mRNA decay rate (Coller)	0.02	2.4E-01	1.3E-01	0.02	2.4E-01	1.3E-01
Protein per mRNA	−0.01	5.9E-01	3.0E-01	−0.01	5.9E-01	3.0E-01
Ribosome occupancy	0.00	9.6E-01	4.9E-01	0.00	9.6E-01	4.8E-01
(B) Based on UTR annotations by Steinmetz and colleagues						
	Spearman			Kendall		
	*ρ*	*P* value	Permutation *P* value	*τ*	*P* value	Permutation *P* value
mRNA decay rate (Cramer 1)	0.01	5.2E-01	2.7E-01	0.01	5.2E-01	2.7E-01
mRNA decay rate (Cramer 2)	0.00	8.9E-01	4.4E-01	0.00	8.9E-01	4.5E-01
mRNA decay rate (Gresham)	0.01	6.4E-01	3.3E-01	0.01	6.4E-01	3.2E-01
mRNA decay rate (Coller)	0.01	4.5E-01	2.3E-01	0.01	4.5E-01	2.3E-01
Protein per mRNA	0.01	6.9E-01	3.4E-01	0.01	6.9E-01	3.4E-01
Ribosome occupancy	0.00	9.5E-01	4.8E-01	0.00	9.5E-01	4.7E-01

(A) Spearman’s and Kendall’s correlation coefficients to assess an association between the presence/absence of hexanucleotide sequences corresponding to the inhibitory codon pairs in 3’ UTR regions. The UTR annotations are based on a study by Snyder and colleagues [17]. *P* values obtained according to Kim [16] and those based on permutation tests are shown. (B) Same as (A) but for UTR annotations based on a study by Steinmetz and colleagues [18]

In sum, the results are largely consistent with the inhibitory codon pairs affecting mRNA decay primarily via its effects on translation elongation kinetics.

### The inhibitory codon pairs cannot fully explain the association of codon optimality with mRNA decay

**Fig. 2:**
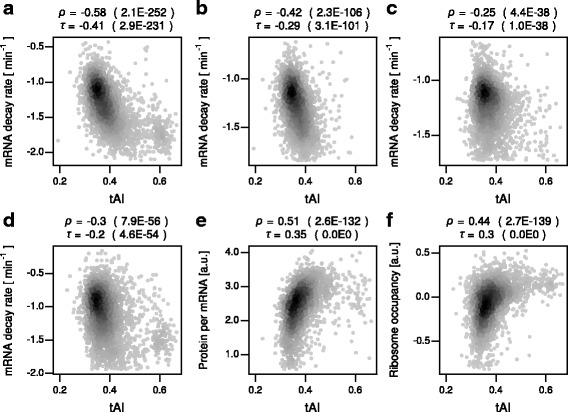
Associations of codon optimality with mRNA decay rate and translation efficiency for genes lacking the inhibitory codon pairs. **a** Scatterplot comparing tAI, a metric of codon optimality, and mRNA decay rate in the “Cramer 1” data (in log10 scale). Shown on the top are the Spearman’s and Kendall’s correlation coefficients and *P* values (parenthesis). **b** Same as (**a**) but for the “Cramer 2” data. **c** Same as (**a**) but for the “Gresham” data. **d** Same as (**a**) but for the “Coller” data. **e** Same as (**a**) but for protein abundance per mRNA. **f** Same as (**a**) but for ribosome occupancy

The inhibitory codon pairs consist of ten types of codons (Table 1), all of which are classified as nonoptimal based on tAI (Additional file 5: Table S5) [19, 20]. Therefore, one possibility is that the inhibitory codon pairs could explain the association of codon optimality with mRNA decay rates [11]. Two observations argue that this is unlikely to be the case. First, for genes lacking the inhibitory codon pairs, codon optimality was still significantly associated with mRNA decay rates (Fig. 2a-d). Second, there was a significant partial correlation of codon optimality with mRNA decay rates even when the analysis was individually or simultaneously controlled for GC content, the content of inhibitory pairs, dipeptide content, and coding length (Table 5 and Additional file 6: Table S6).

**Table 5:** Test for associations of codon optimality with mRNA decay rate, protein per mRNA, and ribosome occupancy

(A) Correlation						
	Spearman			Kendall		
	*ρ*	*P* value	Permutation *P* value	*τ*	*P* value	Permutation *P* value
mRNA decay rate (Cramer 1)	−0.59	0.0E + 00	1.0E-04	−0.42	0.0E + 00	1.0E-04
mRNA decay rate (Cramer 2)	−0.45	7.5E-185	1.0E-04	−0.31	4.6E-176	1.0E-04
mRNA decay rate (Gresham)	−0.24	7.4E-56	1.0E-04	−0.17	1.2E-56	1.0E-04
mRNA decay rate (Coller)	−0.29	1.9E-71	1.0E-04	−0.19	9.7E-70	1.0E-04
Protein per mRNA	0.56	1.6E-246	1.0E-04	0.39	0.0E + 00	1.0E-04
Ribosome occupancy	0.45	5.6E-224	1.0E-04	0.32	0.0E + 00	1.0E-04
(B) Partial correlation controlled for the fraction of the inhibitory codon pairs						
	Spearman			Kendall		
	*ρ*	*P* value	Permutation *P* value	*τ*	*P* value	Permutation *P* value
mRNA decay rate (Cramer 1)	−0.49	9.7E-243	1.0E-04	−0.34	1.6E-233	1.0E-04
mRNA decay rate (Cramer 2)	−0.37	1.9E-125	1.0E-04	−0.25	7.0E-117	1.0E-04
mRNA decay rate (Gresham)	−0.19	6.3E-35	1.0E-04	−0.11	2.9E-27	1.0E-04
mRNA decay rate (Coller)	−0.23	6.1E-47	1.0E-04	−0.16	5.2E-47	1.0E-04
Protein per mRNA	0.49	4.2E-181	1.0E-04	0.33	2.3E-167	1.0E-04
Ribosome occupancy	0.46	1.4E-230	1.0E-04	0.27	2.5E-165	1.0E-04
(C) Partial correlation controlled for the presence/absence of the inhibitory codon pairs						
	Spearman			Kendall		
	*ρ*	*P* value	Permutation *P* value	*τ*	*P* value	Permutation *P* value
mRNA decay rate (Cramer 1)	−0.50	9.4E-254	1.0E-04	−0.34	3.3E-234	1.0E-04
mRNA decay rate (Cramer 2)	−0.38	7.4E-132	1.0E-04	−0.25	3.1E-114	1.0E-04
mRNA decay rate (Gresham)	−0.20	4.2E-37	1.0E-04	−0.11	2.5E-25	1.0E-04
mRNA decay rate (Coller)	−0.24	1.7E-51	1.0E-04	−0.16	6.7E-49	1.0E-04
Protein per mRNA	0.49	4.2E-186	1.0E-04	0.33	1.4E-164	1.0E-04
Ribosome occupancy	0.46	2.2E-235	1.0E-04	0.27	1.9E-158	1.0E-04

(A) Spearman’s and Kendall’s correlation coefficients to assess an association between codon optimality and various gene expression variables. *P* values obtained according to Kim [16] and those based on permutation tests are shown. (B) Same as (A) but for partial correlation coefficients controlled for GC content, the fraction of the inhibitory codon pairs, dipeptide content, and coding length. (C) Same as (A) but for partial correlation coefficients controlled for GC content, the presence/absence of the inhibitory codon pairs, dipeptide content, and coding length

To examine further whether the inhibitory codon pairs can explain effects of codon optimality on mRNA decay rates, we took advantage of synonymous reporter systems used in the previous studies that suggested a mechanistic link between codon optimality and mRNA stability [4, 9, 21–23]. Specifically, we compiled and analyzed the content of the inhibitory codon pairs in sequences of reporter constructs used in the studies (Table 6). The analyses led to the following two points, which imply that the inhibitory codon pairs can promote mRNA instability but cannot explain the relationship between codon optimality and mRNA stability. First, when comparisons are made within a synonymous group, the higher the content of the inhibitory codon pairs, the faster mRNA decay. Second, there are multiple examples where synonymous transcripts that differ in codon optimality but not in the fraction of the inhibitory codon pairs exhibit different mRNA decay rates. The latter point is particularly important because, if the effect of codon optimality were solely due to the inhibitory codon pairs, the synonymous transcripts lacking the inhibitory codon pairs would show similar mRNA decay rates.

**Table 6:** Number of the inhibitory codon pairs in reporter systems

Plasmid name	Gene name	Number of optimal codons	Fraction of optimal codons	Number of inhibitory pairs	Fraction of inhibitory pairs	mRNA decay	Reference
YEpR5	*PGK1*	383	0.923	0	0.0E + 00	Slowest^b^	[21]
YEpR6		242	0.583	3	7.2E-03		
YEpR7		242	0.583	3	7.2E-03		
YEpR8		242	0.583	3	7.2E-03		
YEpR9		242	0.583	3	7.2E-03		
YEpR10		236	0.569	3	7.2E-03	Fastest^b^	
pJC672	Synthetic	58	0.983	0	0.0E + 00	Slow	[4]
pJC673		0	0.000	3	5.2E-02	Fast	
pJC667	*LSM8*	102	0.936	0	0.0E + 00	Slow	[4]
pJC663		48	0.440	0	0.0E + 00	Fast	
pJC716	*HIS3*	208	0.945	0	0.0E + 00	Slowest	[4]
pJC712		96	0.436	1	4.6E-03		
pJC711		1	0.005	3	1.4E-02	Fastest	
NA^a^	*HIS3*	216	0.952	0	0.0E + 00	Slowest	[9]
NA^a^		199	0.877	0	0.0E + 00		
NA^a^		178	0.784	1	4.4E-03		
NA^a^		156	0.687	1	4.4E-03		
NA^a^		135	0.595	1	4.4E-03		
NA^a^		114	0.502	1	4.4E-03		
NA^a^		93	0.410	1	4.4E-03		
NA^a^		72	0.317	1	4.4E-03		
NA^a^		50	0.220	1	4.4E-03		
NA^a^		29	0.128	1	4.4E-03		
NA^a^		9	0.040	3	1.3E-02	Fastest	

^a^NA, not applicable

^b^The effects on mRNA decay rate have been suggested based on mRNA abundance

Overall, these results are consistent with the idea that codon optimality affects mRNA stability at least in part independently of the inhibitory codon pairs.

### The inhibitory codon pairs do not show position effects

**Fig. 3:**
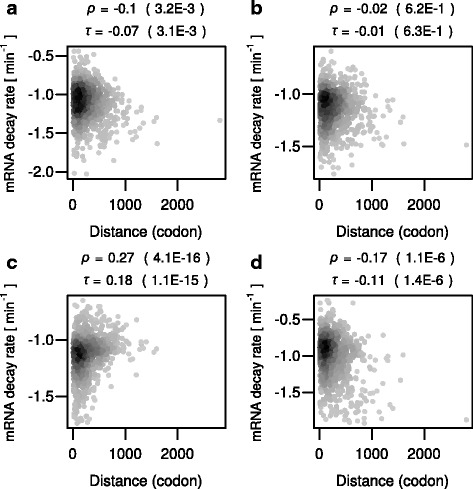
Lack of position effect of the inhibitory codon pairs. **a** Scatterplot comparing mRNA decay rate in the “Cramer 1” data (in log10 scale) and distances of the inhibitory codon pairs from the start codons contained by the mRNAs. Shown on the top are the Spearman’s and Kendall’s correlation coefficients and *P* values (parenthesis). **b** Same as (**a**) but for the “Cramer 2” data. **c** Same as (**a**) but for the “Gresham” data. **d** Same as (**a**) but for the “Coller” data

A previous reporter-based study has shown that a stretch of nonoptimal codons exhibits an increasing destabilizing effect on mRNA with an increasing distance from the start codon [9]. Based on the assumption that the stretch of the nonoptimal codons causes ribosome queuing along the upstream region, the observation was interpreted to suggest that the higher the number of slow ribosomes on a transcript the less stable the transcript [9]. To examine whether the inhibitory codon pairs have a similar property, we computed Spearman’s and Kendall’s correlation coefficients between distances of the inhibitory codon pairs from the start codon and mRNA decay rates in the genome-wide RNA kinetic data. In this analysis, we focused on 1017 ORFs that contain one and only one of the inhibitory codon pairs. If the inhibitory codon pairs had a stronger effect with an increasing distance from the start codon, the distances would be positively correlated with mRNA decay rates. However, we did not observe consistent correlations across the datasets (Fig. 3). This suggests that the inhibitory codon pairs are unlikely to cause a long-range ribosome queuing in upstream regions of natural endogenous mRNAs.

### Codon optimality, adjacent codon pairs, and translation efficiency

It has been shown that codon optimality and the inhibitory codon pairs are associated with translation efficiency [11, 24]. However, another study did not find a correlation between codon optimality and translation efficiency [25].

To reassess this issue, we selected recently published genome-scale data and examined the association between codon optimality and translation efficiency. Translation efficiency, defined as the rate of protein synthesis per mRNA, can be obtained by various methods [26]. One among them is to measure protein abundance and normalize it against mRNA abundance. This is based on the assumption that the majority of proteins are stable and that protein abundance is largely determined by mRNA abundance and protein synthesis rates, which is consistent with a recent protein half-life measurement in *S. cerevisiae* [27]. Another is to use ribosome occupancy on mRNA as a proxy for protein synthesis rates. This is based on the assumption that the majority of mRNA-bound ribosomes are actively engaged in translation, which is consistent with a recent observation in growing *S. cerevisiae* cells that ribosome occupancy is highly correlated with the rate of translation initiation [28].

**Fig. 4:**
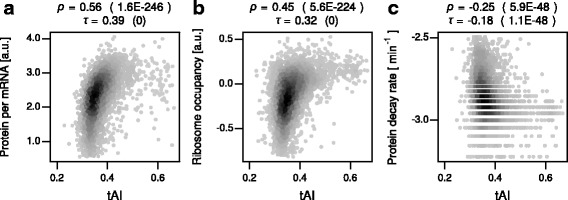
Associations of codon optimality with protein synthesis and decay. **a** Scatterplot comparing tAI, a metric of codon optimality, and protein abundance per mRNA (in log10 scale). Shown on the top are the Spearman’s and Kendall’s correlation coefficients and *P* values (parenthesis). **b** Same as (**a**) but for ribosome occupancy. **c** Same as (**a**) but for protein decay rates

For our analyses, we selected recent quantitative proteomic data by Mann and colleagues [29], which was used in the previous study by Grayhack, Fields, and colleagues [11], as well as mRNA-seq and ribosome profiling data by Weinberg and colleagues [28]. The ribosome profiling data was chosen for two reasons. First, the protocol used for mRNA quantification in the studies does not involve poly(A) enrichment, which could result in 3’ bias, an overestimation of mRNA abundance of short mRNAs, and thus an underestimation of ribosome occupancy of short mRNAs [28]. Second, the protocol used for quantification of ribosome-protected RNA fragments does not involve cycloheximide treatment, which could introduce multiple artifacts. Using these datasets, we observed that translation efficiency in both metrics was positively correlated with codon optimality (Fig. 4a, b, and Additional file 2: Table S2).

We then reexamined the association between the inhibitory codon pairs and translation efficiency in the selected data. For this purpose, we computed Spearman’s and Kendall’s correlation and partial correlation coefficients controlling for the same set of covariates as we used for the analysis of mRNA synthesis/decay rates. The analysis led to the following observations, which is consistent with the previous study [11]. First, the fraction of the inhibitory codon pairs as well as the binary indicator of the presence thereof was associated with low protein abundance per mRNA as well as with low ribosome occupancy (Fig. 1i, j, Table 2A, B, and Additional file 3: Table S3). Second, the association remained significant when the analysis was individually controlled for GC content, codon optimality, dinucleotide content, and coding lengths (Additional file 3: Table S3). Third, the association still remained significant when the analysis was controlled for all the covariates (Table 2C and D).

To assess contributions from the hexanucleotide sequences corresponding to the inhibitory codon pairs, we also examined associations of the sequences in +1 and +2 frames and in 3’ UTRs with translation efficiency. The analyses led to the following three points. First, neither inhibitory codon pairs in the +1 frame nor those in the +2 frame were consistently associated with protein abundance per mRNA (Table 3 and Additional file 4: Table S4). Second, although the content of the inhibitory codon pairs in the +1 and +2 frames were consistently associated with low ribosome occupancy (Additional file 4: Table S4), the association became inconsistent when the covariates were controlled for (Table 3 and Additional file 4: Table S4). Third, the hexanucleotide sequences in 3’ UTRs were not associated with protein abundance per mRNA or ribosome occupancy (Table 4).

Overall, these analyses confirmed a positive correlation between codon optimality and translation efficiency as well as an association between the inhibitory codon pairs and low translation efficiency, which is at least in part independent of effects of nucleotide content, codon optimality, the content of encoded dipeptides, coding lengths, and corresponding hexanucleotide sequences. The results are consistent with the inhibitory codon pairs affecting protein synthesis primarily via its effects on translation elongation kinetics.

### The inhibitory codon pairs cannot fully explain the association of codon optimality with translation efficiency

Having confirmed the associations of codon optimality and the inhibitory codon pairs with translation efficiency, we next examined the possibility that the inhibitory codon pairs can explain the association of codon optimality with translation efficiency. As was seen for mRNA decay rates, two observations argue that this is unlikely to be the case. First, for genes lacking the inhibitory codon pairs, codon optimality was significantly associated with the both metrics of translation efficiency, protein abundance per mRNA (Fig. 2e) and ribosome occupancy (Fig. 2f). Second, there was a significant partial correlation of codon optimality with mRNA decay rates and translation efficiency even when the analysis was individually or simultaneously controlled for GC content, the content of inhibitory pairs, dipeptide content, and coding length (Table 5 and Additional file 6: Table S6).

### Codon optimality, adjacent codon pairs, and protein stability

Translation kinetics can affect co-translational protein folding, which can, in turn, affect protein function and stability. We thus examined associations between codon optimality or adjacent codon pair content and protein decay rates. For this analysis, we used a genome-wide protein half-life measurement by Walther and colleagues [27]. The data was generated via metabolic labeling and thus likely to be more accurate than those generated via methods using translation inhibitors. The analysis led to the following observations. First, codon optimality was negatively correlated with protein decay rates (Fig. 4c and Additional file 2: Table S2). Second, the inhibitory codon pairs were significantly associated with fast protein decay rates (Fig. 1k, Table 2A, and B). Third, however, the association between the inhibitory codon pairs and protein instability became insignificant when tAI was controlled for (Additional file 3: Table S3) and insignificant or borderline significant when all covariates were controlled for (Table 2C and D). These results suggest that the inhibitory codon pairs are unlikely to be directly linked to protein stability.

### Analysis of all possible 3721 adjacent codon pairs

The above analyses are focused on 17 inhibitory codon pairs that have been experimentally characterized previously. However, a recent computational analysis suggests that other codon pairs can be inhibitory to translation [30]. To examine relationships between the adjacent codon pairs and mRNA stability in an unbiased manner, we computed Spearman’s and Kendall’s correlation coefficients between the fraction of each of the possible 3721 codon pairs and mRNA decay rates in the four datasets (Additional file 7: Figure S1 and Additional file 8: Figure S2). We then compared the correlation coefficients between the 17 inhibitory codon pairs and 3704 other pairs. We note that the minimum number of ORFs containing a given codon pair with a measured decay rate was as small as eight, which is for the CGA di-codon, one of the inhibitory codon pairs, in the “Cramer” data. The analysis led to the following two points.

First, in three of the four datasets, the correlations with mRNA decay rates are higher for the inhibitory codon pairs than for other pairs, which is roughly consistent with the association of the inhibitory codon pairs with fast mRNA decay (based on Spearman’s correlations: Wilcoxon rank sum test *P* = 1.0E-6, 3.8E-8, 8.5E-1, and 1.6E-9 for the “Cramer 1”, “Cramer 2”, “Gresham”, and “Coller” data, respectively; based on Kendall’s correlations: *P* = 7.0E-7, 2.0E-8, 7.9E-1, and 1.4E-9 for the “Cramer 1”, “Cramer 2”, “Gresham”, and “Coller” data, respectively) (Additional file 7: Figure S1 and Additional file 8: Figure S2).

Second, for a substantial number of codon pairs other than the 17 inhibitory codon pairs, their content showed significant positive correlations with mRNA decay rates, which may be consistent with those codon pairs acting inhibitory on gene expression as has been suggested in a recent study (Additional file 7: Figure S1 and Additional file 8: Figure S2) [30].

Overall, the results are roughly consistent with the association of the inhibitory codon pairs with fast mRNA decay. However, they also suggest that correlation analyses of individual codon pairs and measured gene expression variables are highly susceptible to experimental noise and/or other confounding factors.

## Discussion

We have analyzed relationships between adjacent codon pairs and aspects of gene expression in *S. cerevisiae*. Our results suggest an association of the inhibitory codon pairs with fast rates of mRNA decay. The association is not solely due to GC content, the content of individual codons, the content of encoded dipeptides, or coding lengths. Moreover, there is no consistent association of the hexanucleotide sequences corresponding to the inhibitory codon pairs in shifted frames or in non-coding regions with mRNA decay rates, which is largely consistent with the inhibitory codon pairs affecting mRNA stability at least in part via their effects on translation elongation kinetics. Although we also observed associations of the inhibitory codon pairs with mRNA synthesis rates and protein instability, they could be attributed to bias in the individual constituent codons.

It has been speculated that adjacent codon pairs rather than individual codons may largely underlie the relationship between codon optimality and translation efficiency [11]. Our results suggest that neither the effects of codon optimality on translation efficiency nor those on mRNA stability can be explained by the 17 inhibitory codon pairs. This observation is more consistent with the model that a sum of subtle effects from individual codons can exert a large impact on gene expression [4]. However, it is still possible that codon pairs rather than individual codons largely define the relationship of codon composition with translation efficiency and mRNA stability since there are likely to be other inhibitory codon pairs than those in the high confidence set obtained by the previous study [11, 30].

The genome-wide associations of codon optimality and the inhibitory codon pairs with mRNA stability and translation efficiency observed by this work and other studies suggest two non-mutually exclusive possibilities. The first possibility is that direct mechanistic links largely underlie the association of the codon composition, which can affect speeds and/or accuracy of translation elongation, with mRNA stability and translation efficiency. Several non-mutually exclusive mechanisms can link slow/inaccurate translation elongation to fast mRNA decay rates and/or low protein synthesis rates. First, slow translation elongation can cause ribosome queuing in upstream regions, which could, in turn, interfere with translation initiation [31, 32]. Second, slow translation elongation may cause ribosome drop-off. Consistent with this idea, the CGA di-codon, one of the inhibitory codon pairs, has been linked to a quality control mechanism that can detect stalled ribosomes and cause abortion of translation [33]. Third, slow translation elongation may be sensed by a mechanism that can modulate translation initiation rates. Indeed, a recent study in *S. cerevisiae* has raised the possibility that the DEAD-box protein Dhh1 might play a central role in such a mechanism [9]. Fourth, nonoptimal codons and/or the inhibitory codon pairs may result in translation repression and mRNA instability by compromising translation fidelity. For example, it is possible that nonoptimal codons and/or the inhibitory codon pairs increase erroneous translation frameshifts. Although there is no overlap between the 17 inhibitory codon pairs and previously identified frameshift-inducing sequences [34, 35], it remains to be determined whether any of the inhibitory codon pairs tend to introduce translation frameshifting. In most cases, translation frameshifts would result in a premature translation termination event at a stop codon in the incorrect frame, which would in turn cause repression of translation initiation and nonsense-mediated mRNA decay (NMD). Such a mechanism can partly underlie the association of nonoptimal codons and inhibitory codon pairs with mRNA instability. Indeed, a recent study suggests that mRNAs with high content of nonoptimal codons tend to undergo nonfunctional translation frameshifts and, subsequently, NMD [36].

The second possibility is that the genome-wide associations between codon composition, translation efficiency, and mRNA stability are largely due to co-evolution rather than to a mechanistic link, the latter of which has been suggested by studies using artificial reporter systems. That is, nonoptimal codons and the inhibitory codon pairs may be simply avoided in natural endogenous genes that are highly expressed and efficiently translated. Then, their primary function may be to modulate local translation elongation speed and thereby regulate other processes, such as co-translational folding, which may be largely restricted to a situation where slow ribosomes do not negatively impact overall translation efficiency. Consistent with this view, some studies in unicellular organisms suggest that under physiological conditions translation initiation but not translation elongation mainly defines the rate of protein synthesis [37, 38]. Moreover, another study did not find corresponding changes in translation efficiency upon genetic manipulation of tRNA and thus codon optimality [39]. Clearly, further investigation will be needed to rigorously evaluate these two possibilities concerning the codon-mediated gene regulation.

## Conclusions

This study suggests genome-scale associations of the inhibitory codon pairs with mRNA decay and translation efficiency, which, in turn, suggest another layer of complexity in the codon-mediated gene regulation. An important future goal will be to understand whether and how the inhibitory codon pairs mechanistically inhibit protein synthesis and elicit mRNA instability.

## Methods

### Data source

Coding sequences and annotations of *S. cerevisiae* (version R64-1-1) were obtained from the Saccharomyces genome database [40]. mRNA synthesis and decay rates were taken from previous studies by Cramer and colleagues [12, 13], by Gresham and colleagues [14], and by Coller and colleagues [4]. mRNA and protein abundance data were taken from previous studies by Ito and colleagues and by Mann and colleagues [29], respectively. Ribosome occupancy was taken from a previous study by Weinberg and colleagues [28]. Protein decay rate was taken from a previous study by Walther and colleague [27]. UTR annotations were taken from previous studies by Snyder and colleagues [17] and by Steinmetz and colleagues [18]. As a metric of optimality of each codon, we used the “relative adaptiveness value” for the tRNA adaptation index [19], also known as classical translation efficiency (cTE) [20]. The relative adaptiveness values are based on tRNA gene copy numbers and selective constraints on the efficiency of codon-anticodon coupling. Weights to represent the constraints are optimized based on gene expression data [19]. In *S. cerevisiae*, the relative adaptiveness values have been shown to correlate positively with translation elongation speeds at individual codons as assessed by ribosome profiling [28, 41]. We took the relative adaptiveness values from a previous study by Tuller and colleagues [24] and computed gene-wise average values (tAI) using the codonR program developed by dos Reis and colleagues [19]. Classification of optimal and nonoptimal codons was taken from a previous study by Frydman and colleagues [20].

### Data filtering and processing

Out of all 6717 annotated ORFs in *S. cerevisiae*, we included all 4879 nuclear-encoded ORFs that are annotated as “verified” (Additional file 9: Table S7) [40]. We used “molecule per minute per cell” and “per minute” as units of mRNA synthesis rates and rates of mRNA/protein decay, respectively. We computed protein abundance per mRNA using proteomic data by Mann and colleagues [29] and mRNA quantification data by Ito and colleagues [42].

### Statistical analysis and graphics

All statistical analyses were performed using R [43]. The cor.test() function in the base package was used to calculate Spearman’s and Kendall’s correlation coefficients. The pcor() function in the ppcor package [16] was used to calculate partial correlation coefficients. The boxplot() function was used to draw boxplots. The heatscatter() function in the LSD package was used to draw scatterplots. The lm() function in the base package was used to build linear regression models. The bptest() function in the lmtest package was used to perform the studentized Breusch-Pagan test.

### Calculation of partial correlation coefficients

To examine associations of the content of inhibitory codon pairs with various gene expression variables controlling for covariates, we first attempted to use multiple linear regression models with exclusion of outliers and logarithmic transformation of skewed variables. However, we found that the models failed to satisfy the assumption of residual homogeneity (see below). We therefore chose to use non-parametric methods throughout the study.

We computed Spearman’s and Kendall’s partial correlation coefficients as described previously [16]. Briefly, we let *X* be a vector of *p* random variables and *c*_*ij*_ be the covariance between two random variables *x*_*i*_ and *x*_*j*_ (1 ≤ *i*, *j* ≤ *p*). We denote the covariance matrix of *X* as *C*_*X*_, the inverse matrix of *C*_*X*_ as *D*_*X*_, and the (*i*, *j*) element of *D*_*X*_ as *d*_*ij*_. We then let *X*_*S*_ be a vector that contains all elements of *X* except *x*_*i*_ and *x*_*j*_. The partial correlation of *x*_*i*_ and *x*_*j*_ given the vector *X*_*S*_ is

$$ {r}_{ij\Big| S} = -\frac{d_{ij}}{\sqrt{d_{ii}}\sqrt{d_{jj}}} $$

Spearman’s partial correlation coefficients were calculated by the Pearson’s method using rank-transformed variables. The Pearson’s and Kendall’s covariance matrices were constructed as follows. Let *x*_*ik*_ be the *k*-th observation for the *i*-th variable *x*_*i*_. The Pearson’s covariance matrix is the matrix whose (*i*, *j*) element is the covariance

$$ {c}_{i j}=\frac{1}{n}{\displaystyle \sum_{k=1}^n}\left({x}_{i k}-{\mu}_i\right)\left({x}_{j k}-{\mu}_j\right) $$

where *n* is the number of observations and *μ*_i_ is the expected value of the *i*-th variable. The Kendall’s covariance matrix is the matrix whose (*i, j*) element is the covariance

$$ {c}_{ij}={\displaystyle \sum_{k=1}^n}{\displaystyle \sum_{l=1}^n} sign\left({x}_{ik}-{x}_{il}\right)\  sign\left({x}_{jk}-{x}_{jl}\right) $$

Note that *sign*(*x*) = 1, 0, − 1 as *x* > 0, = 0, < 0.

We computed *P* values by previously described methods as implemented in the pcor() function in the R ppcor package [16] as well as by permutation tests. To obtain permutation *P* values, we randomly permuted the predictor variables and computed correlation coefficients. We repeated the procedure for 10000 times and computed a permutation *P* value as *(B + 1)/(N + 1),* where *N* is the number of permutations. *B* represents the number of events where the permutation correlation coefficient exceeds the empirically observed value.

### Multiple linear regression models

To build multiple linear regression models, we first log-transformed all variables except the fraction of inhibitory codon pairs, the presence/absence of inhibitory codon pairs, and the fraction of dipeptides encoded by the inhibitory codon pairs. To avoid effects of extreme outliers, we excluded values outside 1.5 times interquartile range. We then performed least square linear regression using the lm() function in the R base package. The resultant estimates for intercepts and slopes can be found in Additional file 10: Table S8. We assessed the assumption of homoscedasticity of errors using the studentized Breusch-Pagan test as implemented in the bptest() function in the R lmtest package. The tests suggest that the assumption was violated (*P* < 0.05) for all models (Additional file 11: Table S9).

## Additional files

Additional file 1: Table S1. Number of genes containing the inhibitory codon pairs. Shown are the number of genes containing at least one of each of the inhibitory codon pairs, the number of genes containing at least one of the 17 inhibitory codon pairs (“Total number of genes with inhibitory pairs”), and the number of genes for which measurements are available in each dataset. Note that 4879 verified ORFs are considered. (XLSX 40 kb)

Additional file 2: Table S2. Pair-wise correlations between all variables used in this study. Shown are Spearman’s and Kendall’s correlation coefficients (A and C) and *P* values (B and D). See also Additional file 9: Table S7. (XLSX 66 kb)

Additional file 3: Table S3. Test for association of the inhibitory codon pairs with mRNA synthesis/decay rate, protein abundance per mRNA, ribosome occupancy, and protein decay rate. (A) Spearman’s correlation and partial correlation coefficients to assess associations between the fraction of the inhibitory codon pairs and the gene expression variables. *P* values obtained according to Kim [16] and those based on permutation tests are shown. (B) Same as (A) but for Kendall’s correlation coefficients. (C) Same as (A) but for the presence/absence of the inhibitory codon pairs. (D) Same as (B) but for the presence/absence of the inhibitory codon pairs. (XLSX 54 kb)

Additional file 4: Table S4. Test for association of the out-of-frame inhibitory codon pairs with mRNA decay rate, protein abundance per mRNA, and ribosome occupancy. (A) Spearman’s correlation and partial correlation coefficients to assess associations between the fraction of the inhibitory codon pairs in the +1 frame and the gene expression variables. *P* values obtained according to Kim [16] and those based on permutation tests are shown. (B) Same as (A) but for Kendall’s correlation coefficients. (C) Same as (A) but for the presence/absence of the inhibitory codon pairs. (D) Same as (B) but for the presence/absence of the inhibitory codon pairs. (E) Same as (A) but for the +2 frame. (F) Same as (B) but for the +2 frame. (G) Same as (C) but for the +2 frame. (H) Same as (D) but for the +2 frame. (XLSX 57 kb)

Additional file 5: Table S5. Properties of 61 nonstop codons. Shown are corresponding amino acids, relative adaptiveness values for tAI [19, 24], classification of optimal (O) and nonoptimal (N) codons [20], and a binary variable to indicate whether the codon constitutes the inhibitory codon pairs [11]. (XLSX 42 kb)

Additional file 6: Table S6. Test for association of codon optimality with mRNA decay rate, protein abundance per mRNA, and ribosome occupancy. (A) Spearman’s correlation and partial correlation coefficients to assess associations between codon optimality and the gene expression variables. *P* values obtained according to Kim [16] and those based on permutation tests are shown. (B) Same as (A) but for Kendall’s correlation coefficients. (XLSX 44 kb)

Additional file 7: Figure S1. **Figure S1** Analysis of all possible 3721 codon pairs. (A) Plotted are ordered Spearman’s correlation coefficients between the fraction of individual codon pairs and mRNA decay rates in the “Cramer 1” data. The 17 inhibitory codon pairs are labeled. Also shown is the *P* value from Wilcoxon rank sum test with an alternative hypothesis that correlation coefficients are greater for the 17 inhibitory codon pairs than for other pairs. (B) Same as (A) but for the “Cramer 2” data. (C) Same as (A) but for the “Gresham” data. (D) Same as (C) but for the “Coller” data. (PDF 57 kb)

Additional file 8: Figure S2. Analysis of all possible 3721 codon pairs. (A) Plotted are ordered Kendall’s correlation coefficients between the fraction of individual codon pairs and mRNA decay rates in the “Cramer 1” data. The 17 inhibitory codon pairs are labeled. Also shown is the *P* value from Wilcoxon rank sum test with an alternative hypothesis that correlation coefficients are greater for the 17 inhibitory codon pairs than for other pairs. (B) Same as (A) but for the “Cramer 2” data. (C) Same as (A) but for the “Gresham” data. (D) Same as (C) but for the “Coller” data. (PDF 56 kb)

Additional file 9: Table S7. mRNA synthesis/decay rate, protein abundance per mRNA, ribosome occupancy, fraction of inhibitory pairs, presence/absence of inhibitory pairs, GC content, tAI, fraction of dipeptides, and coding length for 4879 genes. (XLSX 1155 kb)

Additional file 10: Table S8. Multivariate linear models. Shown are intercept and slope estimates. (XLSX 44 kb)

Additional file 11: Table S9. Test for heteroscedasticity. Shown are *P* values from studentized Breusch-Pagan tests to assess heteroscedasticity of the residuals of the linear models. (XLSX 46 kb)

## Abbreviations

3’ UTR: Three prime untranslated region
GC content: Guanine-cytosine content
NMD: Nonsense-mediated mRNA decay
tAI: tRNA adaptation index
