# Erratum to: the link between adjacent codon pairs and mRNA stability

**DOI:** 10.1186/s12864-017-4088-5

**Published:** 2017-09-08

**Authors:** Yuriko Harigaya, Roy Parker

**Affiliations:** Department of Chemistry and Biochemistry, Howard Hughes Medical Institute, University of Colorado Boulder, Boulder, CO 80303 USA

## Erratum

After publication of this article [1], the authors noticed two errors:

In Table 3B and D, the column labels should be “Frame 0”, “Frame 1”, and “Frame 2” rather than “Frame 0”, “Frame 0”, and “Frame 0.” (Table 3).

**Table 3:** Test for associations of the out-of-frame inhibitory codon pairs with mRNA decay rate, protein per mRNA, and ribosome occupancy

(A) Spearman’s partial correlation based on the fraction of the inhibitory codon pairs									
	Frame 0			Frame 1			Frame 2		
	*ρ*	*P* value	Perm.	*ρ*	*P* value	Perm.	*ρ*	*P* value	Perm.
			*P* value			*P* value			*P* value
mRNA decay rate (Cramer 1)	0.14	2.1E-18	1.0E-04	0.05	1.0E-03	7.0E-04	0.02	3.4E-01	1.7E-01
mRNA decay rate (Cramer 2)	0.15	1.2E-20	1.0E-04	0.02	2.4E-01	1.3E-01	0.03	3.8E-02	1.8E-02
mRNA decay rate (Gresham)	0.07	9.0E-06	1.0E-04	0.06	2.0E-04	2.0E-04	0.01	5.2E-01	2.6E-01
mRNA decay rate (Coller)	0.06	2.0E-04	1.0E-04	0.02	2.2E-01	1.1E-01	0.03	4.6E-02	2.4E-02
Protein per mRNA	−0.07	4.9E-05	1.0E-04	0.03	8.6E-02	4.3E-02	0.01	7.2E-01	3.6E-01
Ribosome occupancy	−0.06	8.8E-05	1.0E-04	0.02	2.0E-01	9.9E-02	0.01	5.2E-01	2.7E-01
(B) Kendall’s partial correlation based on the fraction of the inhibitory codon pairs									
	Frame 0			Frame 1			Frame 2		
	*τ*	*P* value	Perm.	*τ*	*P* value	Perm.	*τ*	*P* value	Perm.
			*P* value			*P* value			*P* value
mRNA decay rate (Cramer 1)	0.15	2.4E-48	1.0E-04	0.04	6.7E-04	1.0E-04	0.00	7.7E-01	3.8E-01
mRNA decay rate (Cramer 2)	0.16	9.8E-49	1.0E-04	0.02	2.5E-02	9.7E-03	0.02	7.1E-02	3.2E-02
mRNA decay rate (Gresham)	0.10	7.9E-22	1.0E-04	0.08	2.6E-13	1.0E-04	0.03	1.3E-02	4.0E-03
mRNA decay rate (Coller)	0.07	6.2E-10	1.0E-04	0.01	6.1E-01	3.1E-01	0.01	3.8E-01	2.0E-01
Protein per mRNA	−0.11	7.3E-20	1.0E-04	0.01	3.7E-01	1.7E-01	0.01	6.2E-01	3.1E-01
Ribosome occupancy	−0.11	1.3E-30	1.0E-04	−0.02	2.2E-02	3.9E-03	−0.01	4.6E-01	2.0E-01
(C) Spearman’s partial correlation based on the presence/absence of the inhibitory codon pairs									
	Frame 0			Frame 1			Frame 2		
	*ρ*	*P* value	Perm.	*ρ*	*P* value	Perm.	*ρ*	*P* value	Perm.
			*P* value			*P* value			*P* value
mRNA decay rate (Cramer 1)	0.13	2.8E-16	1.0E-04	0.04	1.1E-02	7.3E-03	0.00	8.7E-01	4.3E-01
mRNA decay rate (Cramer 2)	0.14	8.6E-19	1.0E-04	0.01	4.0E-01	2.0E-01	0.01	5.5E-01	2.7E-01
mRNA decay rate (Gresham)	0.07	2.2E-05	1.0E-04	0.06	2.9E-04	3.0E-04	0.01	6.0E-01	3.0E-01
mRNA decay rate (Coller)	0.04	7.5E-03	3.8E-03	0.02	1.7E-01	8.1E-02	0.02	2.7E-01	1.4E-01
Protein per mRNA	−0.07	6.3E-05	1.0E-04	0.03	1.6E-01	8.1E-02	0.00	9.9E-01	4.9E-01
Ribosome occupancy	−0.06	1.6E-05	2.0E-04	0.01	4.1E-01	2.0E-01	0.00	9.6E-01	4.8E-01
(D) Kendall’s partial correlation based on the presence/absence of the inhibitory codon pairs									
	Frame 0			Frame 1			Frame 2		
	*τ*	*P* value	Perm.	*τ*	*P* value	Perm.	*τ*	*P* value	Perm.
			*P* value			*P* value			*P* value
mRNA decay rate (Cramer 1)	0.16	1.1E-50	1.0E-04	0.03	1.2E-03	1.5E-03	0.00	1.0E + 00	4.9E-01
mRNA decay rate (Cramer 2)	0.17	2.7E-57	1.0E-04	0.04	8.8E-04	2.1E-03	0.02	6.1E-02	5.1E-02
mRNA decay rate (Gresham)	0.12	8.3E-29	1.0E-04	0.12	7.0E-31	1.0E-04	0.07	1.4E-10	1.0E-04
mRNA decay rate (Coller)	0.06	5.9E-08	1.0E-04	0.00	9.8E-01	4.9E-01	0.00	6.6E-01	3.5E-01
Protein per mRNA	−0.12	3.2E-23	1.0E-04	0.01	3.2E-01	2.0E-01	0.00	9.6E-01	4.8E-01
Ribosome occupancy	−0.14	3.7E-44	1.0E-04	−0.07	3.4E-11	1.0E-04	−0.05	2.6E-07	1.0E-04

A) Spearman’s partial correlation coefficients controlled for GC content, tAI, dipeptide content, and coding length to assess an association between the fraction of hexanucleotide sequences corresponding to the inhibitory codon pairs in the 0, +1, and +2 frames and various gene expression variables. *P* values obtained according to Kim [16] and those based on permutation tests are shown. (B) Same as (A) but for Kendall’s partial correlation coefficients. (C) Same as (A) but for the presence/absence of the hexanucleotide sequences. (D) Same as (B) but for the presence/absence of the hexanucleotide sequences

A corrected version of Table 3 is included with this Erratum.

In the “Calculation of partial correlation coefficients” section in Methods, the expression of the Pearson’s covariance matrix is incorrect. The denominator should be n − 1 instead of n. In the corrected version, the authors clarify the computational implementation used. Also, the authors now follow the convention where random variables are expressed in uppercase letters.

A corrected version of this follows below (references included in the revised portion are referring to the original article):

## Methods

### Calculation of partial correlation coefficients

To examine associations of the content of inhibitory codon pairs with various gene expression variables controlling for covariates, we first attempted to use multiple linear regression models with exclusion of outliers and logarithmic transformation of skewed variables. However, we found that the models failed to satisfy the assumption of residual homogeneity (see below). We therefore chose to use non-parametric methods throughout the study.

We computed Spearman’s and Kendall’s partial correlation coefficients as described previously [16]. Briefly, we let X be a p-dimensional random vector (X = [X_1_ X_2_ ⋯ X_p_]^T^) and c_ij_ be the covariance between two random variables X_i_ and X_j_ (1 ≤ i , j ≤ p). We denote the covariance matrix of X as C_X_, the inverse covariance matrix as D_X_, and the (i, j) element of D_X_ as d_ij_. We then let X_S_ be a vector that contains all elements of X except X_i_ and X_j_. The partial correlation of X_i_ and X_j_ given the vector X_S_ is

$$ {\mathrm{r}}_{\mathrm{ij}\mid \mathrm{S}}=-\frac{{\mathrm{d}}_{\mathrm{ij}}}{\sqrt{{\mathrm{d}}_{\mathrm{ii}}}\sqrt{{\mathrm{d}}_{\mathrm{jj}}}} $$

The Spearman’s and Kendall’s covariance matrices were constructed as implemented in the cov() function in the R base package [43].

We computed *P* values by previously described methods as implemented in the pcor() function in the R ppcor package [16] as well as by permutation tests. To obtain permutation *P* values, we randomly permuted the predictor variables and computed correlation coefficients. We repeated the procedure for 10,000 times and computed a permutation *P* value as (B + 1)/(N + 1), where N is the number of permutations. B represents the number of events where the permutation correlation coefficient exceeds the empirically observed value.
