# Supplementary figures and images for: The link between adjacent codon pairs and mRNA stability
Source: BMC Genomics. 2017 May 10;18:364. doi: 10.1186/s12864-017-3749-8 (PMC5424319; doi:10.1186/s12864-017-3749-8)

**A**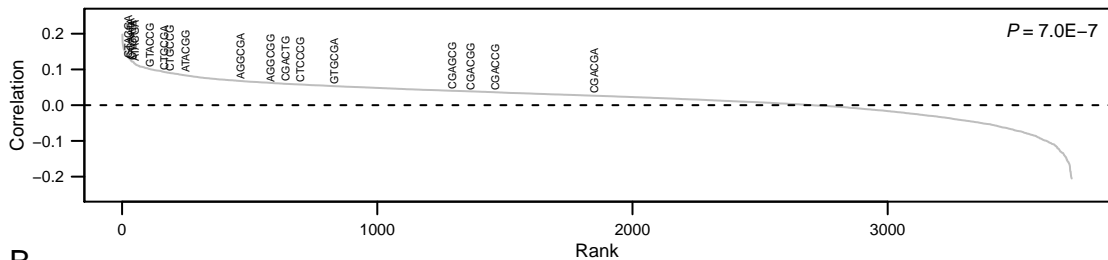**B**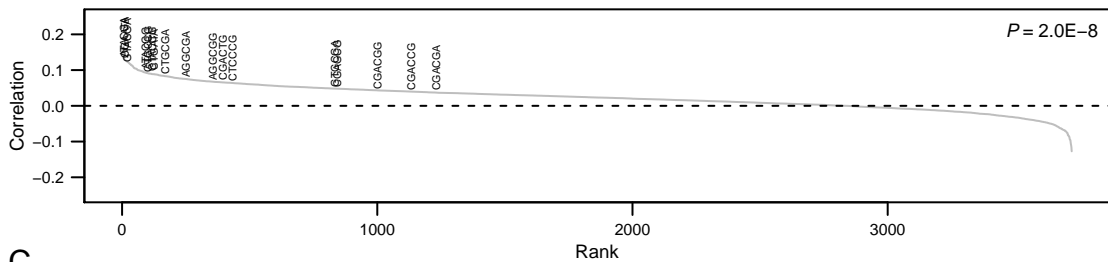**C**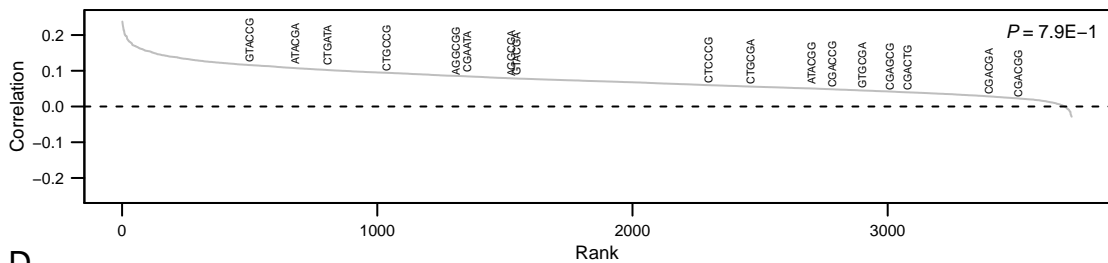**D**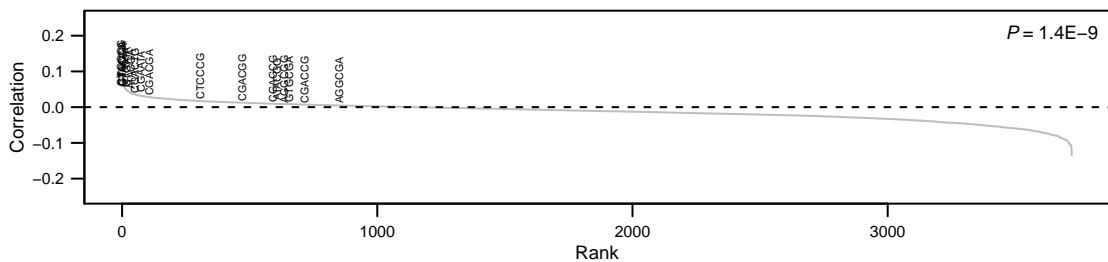

Supplement: Supplementary file 8 — Analysis of all possible 3721 codon pairs. (A) Plotted are ordered Kendall’s correlation coefficients between the fraction of individual codon pairs and mRNA decay rates in the “Cramer 1” data. The 17 inhibitory codon pairs are labeled. Also shown is the P value from Wilcoxon rank sum test with an alternative hypothesis that correlation coefficients are greater for the 17 inhibitory codon pairs than for other pairs. (B) Same as (A) but for the “Cramer 2” data. (C) Same as (A) but for the “Gresham” data. (D) Same as (C) but for the “Coller” data. (PDF 56 kb) [file 12864_2017_3749_MOESM8_ESM.pdf]
