# Supplementary material for: The link between adjacent codon pairs and mRNA stability
Source: BMC Genomics. 2017 May 10;18:364. doi: 10.1186/s12864-017-3749-8 (PMC5424319; doi:10.1186/s12864-017-3749-8)
Supplement: Supplementary file 7 — Figure S1 Analysis of all possible 3721 codon pairs. (A) Plotted are ordered Spearman’s correlation coefficients between the fraction of individual codon pairs and mRNA decay rates in the “Cramer 1” data. The 17 inhibitory codon pairs are labeled. Also shown is the P value from Wilcoxon rank sum test with an alternative hypothesis that correlation coefficients are greater for the 17 inhibitory codon pairs than for other pairs. (B) Same as (A) but for the “Cramer 2” data. (C) Same as (A) but for the “Gresham” data. (D) Same as (C) but for the “Coller” data. (PDF 57 kb) [file 12864_2017_3749_MOESM7_ESM.pdf]

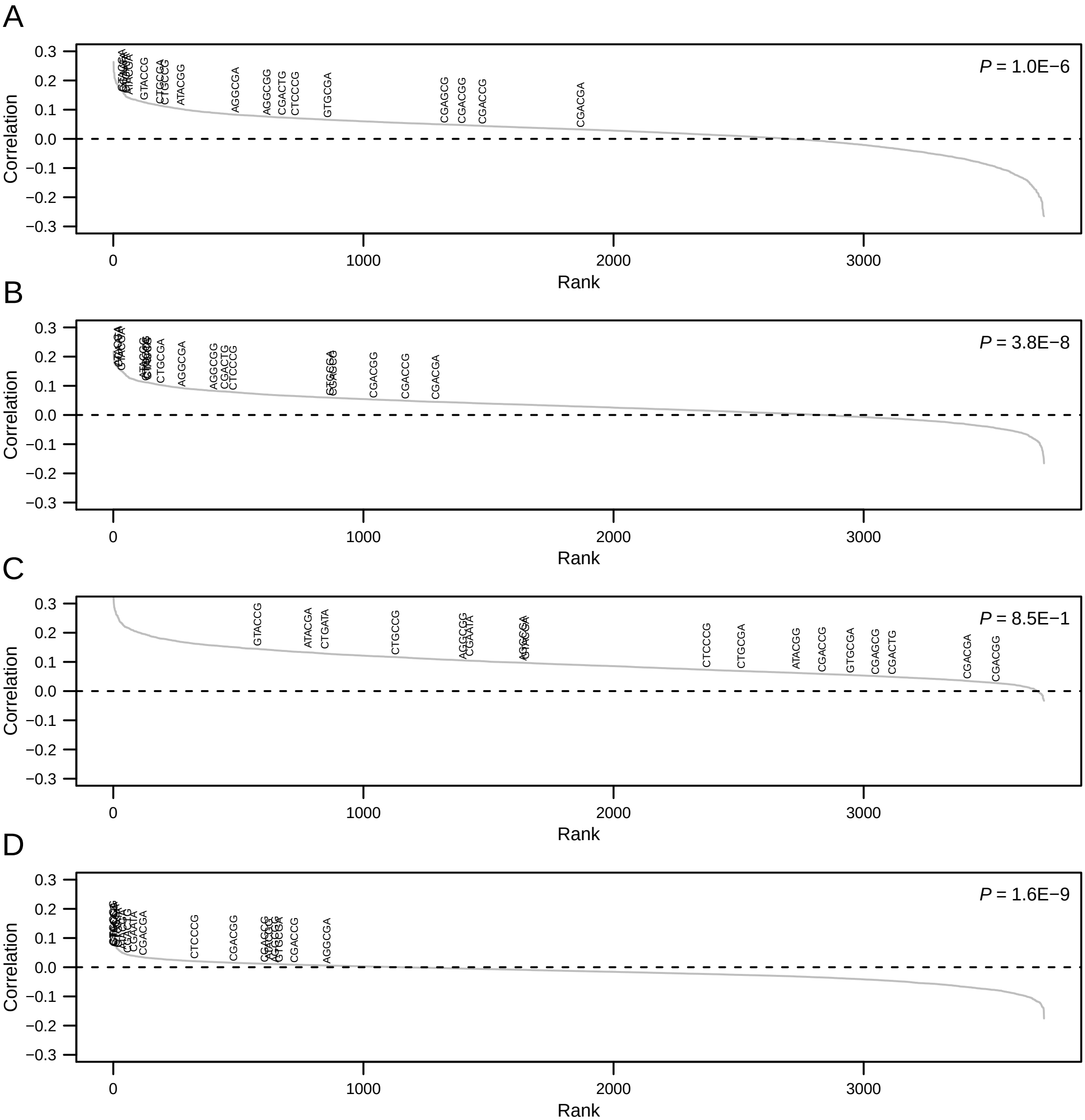
A
P = 1.0E−6
GTACCG
CTGCGA
CTGCCG
ATACGG
AGGCGA
AGGCGG
CGACTG
CTCCCG
GTGCGA
CGAGCG
CGACGG
CGACCG
CGACGA
B
P = 3.8E−8
CTGCGA
AGGCGA
AGGCGG
CGACTG
CTCCCG
CGACGG
CGACCG
CGACGA
C
P = 8.5E−1
GTACCG
ATACGA
CTGATA
CTGCCG
AGGCGG
CGAATA
CTCCCG
CTGCGA
ATACGG
CGACCG
GTGCGA
CGAGCG
CGACTG
CGACGA
CGACGG
D
P = 1.6E−9
CGACGA
CTCCCG
CGACGG
CGACCG
AGGCGA
Correlation
Rank
0
1000
2000
3000
0.3
0.2
0.1
0.0
−0.1
−0.2
−0.3
